# Effects of Souvenaid on plasma micronutrient levels and fatty acid profiles in mild and mild-to-moderate Alzheimer’s disease

**DOI:** 10.1186/s13195-015-0134-1

**Published:** 2015-07-24

**Authors:** Anne Rijpma, Olga Meulenbroek, Anneke M. J. van Hees, John W. C. Sijben, Bruno Vellas, Raj C. Shah, David A. Bennett, Philip Scheltens, Marcel G. M. Olde Rikkert

**Affiliations:** Radboud Alzheimer Center, Department of Geriatric Medicine, Radboud University Medical Center, P.O. Box 9101, 6500 HB Nijmegen, The Netherlands; Nutricia Research, Nutricia Advanced Medical Nutrition, Utrecht, The Netherlands; Gerontopole, INSERM U1027, Toulouse, France; Rush Alzheimer’s Disease Center, Rush University Medical Center, Chicago, IL USA; Alzheimer Center, VU University Medical Center, Amsterdam, The Netherlands

## Abstract

**Introduction:**

Circulating levels of uridine, selenium, vitamins B_12_, E and C, folate, docosahexaenoic acid (DHA) and eicosapentaenoic acid (EPA) have been shown to be lower in patients with Alzheimer’s disease (AD) than in healthy individuals. These low levels may affect disease pathways involved in synapse formation and neural functioning. Here, we investigated whether, and to what extent, circulating levels of micronutrients and fatty acids can be affected by oral supplementation with Souvenaid (containing a specific nutrient combination), using data derived from three randomized clinical trials (RCT) and an open-label extension (OLE) study with follow-up data from 12 to 48 weeks.

**Methods:**

Subjects with mild (RCT1, RCT2) or mild-to-moderate AD (RCT3) received active or control product once daily for 12–24 weeks or active product during the 24-week OLE following RCT2 (*n* = 212–527). Measurements included plasma levels of B vitamins, choline, vitamin E, selenium, uridine and homocysteine and proportions of DHA, EPA and total n-3 long-chain polyunsaturated fatty acids in plasma and erythrocytes. Between-group comparisons were made using *t* tests or non-parametric alternatives.

**Results:**

We found that 12–24-week active product intake increased plasma and/or erythrocyte micronutrients: uridine; choline; selenium; folate; vitamins B_6_, B_12_ and E; and fatty acid levels of DHA and EPA (all *p* < 0.001). In the OLE study, similar levels were reached in former control product/initial active product users, whereas 24-week continued active product intake showed no suggestion of a further increase in nutrient levels.

**Conclusions:**

These data show that circulating levels of nutrients known to be decreased in the AD population can be increased in patients with mild and mild-tomoderate AD by 24–48-week oral supplementation with Souvenaid. In addition, to our knowledge, this is the first report of the effects of sustained dietary intake of uridine monophosphate on plasma uridine levels in humans. Uptake of nutrients is observed within 6 weeks, and a plateau phase is reached for most nutrients during prolonged intake, thus increasing the availability of precursors and cofactors in the circulation that may be used for the formation and function of neuronal membranes and synapses in the brain.

**Electronic supplementary material:**

The online version of this article (doi:10.1186/s13195-015-0134-1) contains supplementary material, which is available to authorized users.

## Introduction

Several disease pathways and risk factors for Alzheimer’s disease (AD) are hypothesized to be affected by nutritional factors, such as reduced neuronal membrane integrity and function, and phospholipid metabolism [1–3]. Correspondingly, epidemiological studies have repeatedly shown the protective and/or risk-reducing effects of nutritional intake on AD [4–6]. In addition, patients with AD are frequently reported to have lower plasma levels of certain nutrients than healthy controls [7–10]. Data derived from our own studies have shown lower plasma levels of uridine, selenium, docosahexaenoic acid (DHA) in patients with mild AD than in healthy age-matched controls [10]. A recent meta-analysis showed lower levels of folate and vitamins A, B_12_, C and E in patients with AD than in healthy controls [7]. Together, these studies suggest a connection between nutrient status and AD. Counteracting any nutritional deficiencies may therefore have a beneficial effect on patients with AD.

The medical food Souvenaid, which contains the specific nutrient combination Fortasyn Connect (both products of Nutricia Advanced Medical Nutrition, Utrecht, the Netherlands), has been designed to address the distinct nutritional needs of patients with AD and thereby ameliorate synapse loss and synaptic dysfunction in AD. The medical food was developed to increase brain levels of specific nutrients to support the process of neuronal membrane formation [11]. In turn, increased brain nutrient levels can stimulate synapse formation to compensate for synapse loss in AD [12, 13]. A prerequisite for potentially increasing brain nutrient levels is that the intervention will indeed raise circulating nutrient levels, thus increasing their availability for neuronal membrane formation.

A number of randomized clinical trials (RCTs) have been performed to investigate the effect of this specific nutrient combination on cognitive function in patients with mild AD [14, 15] (referred to hereinafter as RCT1 and RCT2, respectively) and those with mild-to-moderate AD [16] (referred to hereinafter as RCT3). One open-label extension (OLE) study (extension of RCT2) has been performed with safety as the primary endpoint and memory as an exploratory endpoint. Both trials in drug-naïve patients with mild AD showed improvement in memory domain scores after 12 weeks (RCT1) and 24 weeks (RCT1 and RCT2) [14–16]. The OLE study showed that use of this specific nutrient combination for up to 48 weeks was well tolerated. Furthermore, a significant increase in the exploratory memory outcome was observed in both the active-active and control-active groups from 24 to 48 weeks of use [17]. In the clinical trial in patients with mild-to-moderate AD and on AD medication, no change in cognitive function was found after 24 weeks (RCT3) [18].

Blood samples were taken at baseline and at the end of the study in all RCTs. Here, we investigate whether, and to what extent, circulating levels of micronutrients and fatty acids, among which several are known to be decreased in the AD population, are affected by 12–48-week oral supplementation with Souvenaid in patients with mild and mild-to-moderate AD.

## Methods

Three double-blind, multicenter, controlled RCTs (Souvenir [RCT1], Souvenir II [RCT2] and S-Connect [RCT3]) were performed between 2006 and 2011 to evaluate the effects of the medical food Souvenaid on cognition and memory performance in patients with AD [15, 16, 18]. Subjects who completed the 12-week intervention of RCT1 were invited to participate in a 12-week double-blind extension study. In addition, an RCT2 OLE study was performed between 2010 and 2012 to evaluate longer-term safety of and compliance with Souvenaid [19]. Here we present the results of the analyses of secondary and exploratory plasma micronutrient parameters and fatty acid profiles in plasma, plasma phospholipids and erythrocyte membranes. Some results have been reported previously in publications describing the results of the RCTs, mainly as compliance markers [15, 16, 18, 19]. All trials were registered in the Dutch Trial Register (Souvenir: NTR702; Souvenir II: NTR1975; S-Connect: NTR1683; and OLE: NTR2571).

### Study population

The study population and methodology of the studies have been described in detail previously [15, 16, 18, 19]. Briefly, all studies included men and women ≥50 years of age who were diagnosed with probable AD according to the National Institute of Neurological and Communicative Disorders and Stroke-Alzheimer’s Disease and Related Disorders Association criteria [20] and were either in (1) the mild stage of AD as defined by Mini Mental State Examination (MMSE) scores of 20–26 (RCT1) or ≥20 (RCT2) or (2) the mild-to-moderate stage of AD, defined as an MMSE score of 14–24 inclusive (RCT3). Subjects in RCT1 and RCT2 had to be drug-free for AD medication, whereas subjects in RCT3 were on a stable dose of approved AD medication. Subjects were not allowed to use, within 1–2 months before study participation and during the study, fatty acid–containing supplements (RCT1, RCT2 and RCT3); to consume oily fish more than twice per week (RCT2 and RCT3); to use vitamins B, C and/or E >200 % (RCT1 and RCT2) or >100 % (RCT3) of recommended dietary allowance (RDA) [21, 22]; or to use high-energy and/or high-protein nutritional supplements and/or medical foods (RCT2, 3). At the end of RCT2, all subjects who completed the study were invited to participate in the OLE. Eligibility criteria for the OLE allowed patients to use AD medication and nutritional supplements.

### Study procedures

All subjects were randomly assigned to receive the active or control product once daily for 12 weeks (RCT1) or 24 weeks (RCT1 extension, RCT2 and RCT3), whereas during the OLE study, all subjects received the active product. The active product (Souvenaid) contains the specific nutrient combination Fortasyn Connect (Table 1). The control product is isocaloric and similar in appearance and flavor to the active product, but without Fortasyn Connect. Both study products were available in the form of a 125-ml drink (125 kcal) in vanilla or strawberry flavor (RCT2, RCT3 and OLE) and peach-orange or cappuccino flavor (RCT1).

**Table 1:** Nutritional composition of the study products in amount per daily dose (125 ml)

	Control	Active
Energy	125 kcal	125 kcal
Protein	3.8 g	3.8 g
Carbohydrate	16.5 g	16.5 g
Fat	4.9 g	4.9 g
EPA	0	300 mg
DHA	0	1200 mg
Phospholipids	0	106 mg
Choline	0	400 mg
UMP	0	625 mg
Vitamin E (α-TE)	0	40 mg
Vitamin C	0	80 mg
Selenium	0	60 μg
Vitamin B_12_	0	3 μg
Vitamin B_6_	0	1 mg
Folic acid	0	400 μg

*DHA* docosahexaenoic acid, *EPA* eicosapentaenoic acid, *TE* tocopherol equivalents, *UMP* uridine monophosphate

Study staff, subjects and caregivers were blinded to each subject’s randomized study group allocation throughout all studies, including the extension of RCT1 and the OLE (i.e., blinding for group allocation during RCT2 continued).

**Fig. 1:**
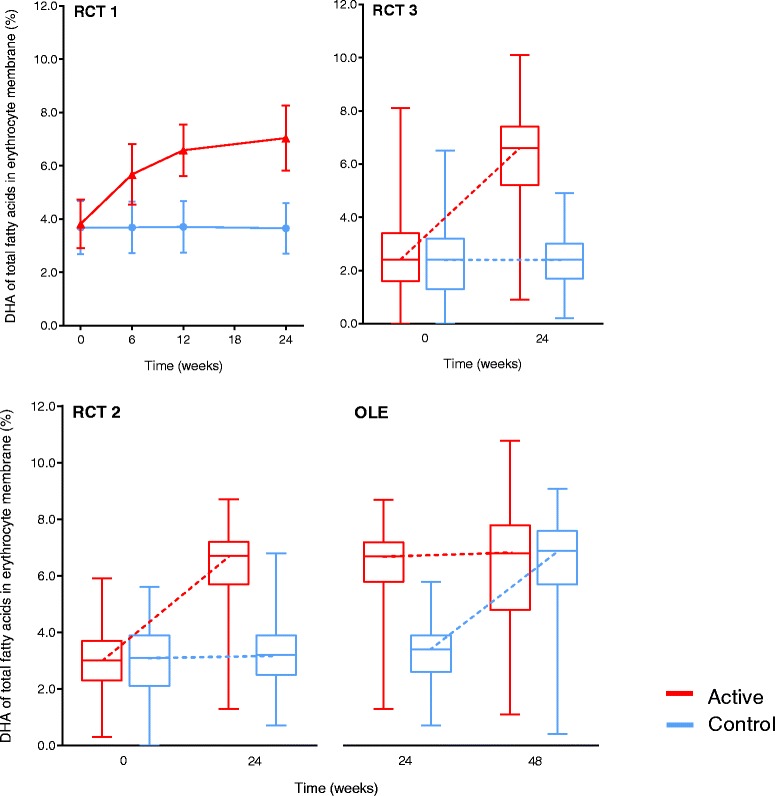
Docosahexaenoic acid (DHA) of total fatty acids in erythrocyte membrane (%) in randomized clinical trial 1 (RCT1), RCT2, RCT3 and the open-label extension (OLE) study in the active and control groups. RCT1, Mean±SD; RCT2,3 and OLE, boxplots show median values (solid horizontal line), 25th-75th percentile values (box outline) and minumum and maximum values (whiskers). Dashed lines connect median values between time points within groups

Outcome parameters for the current analyses were assessed at baseline and, depending on the nutrient, week 24 of RCT1, RCT2 and RCT3 and at week 24 (presented as week 48) of the OLE. The screening or baseline visit for the OLE was combined with the final visit (week 24) of RCT2. In addition, homocysteine (Hcy), vitamin E, uric acid and percentages of DHA, eicosapentaenoic acid (EPA) and docosapentaenoic acid (DPA) of total fatty acids in erythrocyte membrane also were assessed at week 6 and week 12 of RCT1. These are not statistically analyzed in the present article. However, data on percentage DHA of total fatty acids in erythrocyte membrane is shown in Fig. 1.

Written informed consent was obtained from subjects and their caregivers before study participation. The ethics committee of each participating center in each study reviewed and approved the protocol (see Additional file 1). The studies were conducted in accordance with the Declaration of Helsinki, the International Conference on Harmonization Guidelines for Good Clinical Practice as appropriate for nutritional products and the local laws and regulations of the country in which the research was conducted.

### Study parameters

Fasting (RCT1) and non-fasting (RCT2, RCT3 and OLE) venous blood samples were taken to determine plasma levels of folate, Hcy, vitamins B_6_ and B_12_, choline, vitamins D and E, selenium, uridine and fatty acids (including DHA, EPA, DPA and total n-3 long-chain polyunsaturated fatty acids [n-3 LC-PUFA]). In addition, erythrocytes were collected to determine fatty acid (including DHA, EPA, DPA and n-3 LC-PUFA) levels in the erythrocyte membrane. In RCT1, the results in plasma vitamin C analyses varied greatly, preventing meaningful interpretation. For that reason, in RCT2 and RCT3, vitamin C levels were not analyzed. In RCT1, venous blood samples also were taken to determine plasma levels of uracil, uric acid, cytidine, malondialdehyde (MDA), (pre-)albumin, C-reactive protein (CRP), interleukin (IL)-1β, IL-6, IL-10 and 8-isoprostane.

Parameters were assessed in the intention-to-treat (ITT) population or in subgroups, based on the availability of blood samples. The actual number of analyzed samples is indicated in the tables and Fig. 1 for each parameter.

Information on preexisting and new use of medication and nutritional supplements was collected throughout the studies.

### Biochemical analyses

Blood was collected in tubes containing ethylenediaminetetraacetic acid. All samples were centrifuged (1300 × *g*, 15 min, 4 °C), and plasma and erythrocyte aliquots were stored at −70 °C / −80 °C (RCT1, RCT2 and OLE) or at least −20 °C (RCT3) until analysis at a central laboratory. For RCT2 and the OLE, all baseline and 24-week samples were analyzed together as part of RCT2, whereas all 48-week samples were analyzed at the end of the OLE.

Plasma folate and vitamin B_12_ levels were determined using a competitive protein binding ligand assay. Plasma B_6_ levels were measured by performing high-performance liquid chromatography (HPLC). HPLC electrochemical detection of plasma-free choline was performed according to a method adapted from one previously described by Fossati *et al.* [23] and as reported previously [24]. Plasma albumin was determined using a colorimetric kit; plasma selenium levels were measured using graphite furnace atomic absorption spectrometry; and plasma pre-albumin and CRP levels were assessed using turbimetric assays. A microparticle chemiluminescence immunoassay (ARCHITECT assay; Abbott Diagnostics, Lake Forest, IL, USA) was used to determine plasma vitamin D (total 25-hydroxyvitamin D) levels. Plasma vitamin E levels were determined by performing HPLC using fluorometric properties for detection of α-tocopherol by comparison with standard solutions [25]. For the determination of plasma Hcy levels, thiol amino acids (free and protein-bound) were reduced with tri-*n*-butylphosphine. After precipitation with trichloroacetic acid, thiol groups were derivatized with ammonium 7-fluorobenzo-2-oxa-1,3-diazole-4-sulfonate, followed by separation using HPLC with a fluorescence detector [26, 27]. Determination of MDA was based on the thiobarbituric acid and reversed-phase HPLC separation with fluorescence detection [28]. To determine plasma uridine, uracil and cytidine levels, perchloric acid was added to the sample. Uridine, uracil and cytidine were extracted by vortexing the solution, followed by separation from other nucleotides and/or nucleosides using reversed-phase HPLC [29]. The compounds were quantified by measuring absorbance compared with a standard. Uric acid levels in plasma were determined using an enzymatic assay. Plasma levels of cytokines (IL-1β, IL-6 and IL-10) and free 8-isoprostane were measured using, respectively, a commercial, custom-made human Bio-Plex cytokine bead-based immunoassay (Bio-Rad Laboratories, Hercules, CA, USA) and a commercially available enzyme immunoassay (Cayman Chemical, Ann Arbor, MI, USA) according to the manufacturers’ protocols. The fatty acid composition of the total lipid fraction in plasma and erythrocytes was analyzed qualitatively on a gas chromatograph after extraction of the lipids from the plasma and/or erythrocytes and a methylation step [30–33].

### Statistical analyses

Analyses were performed on the ITT population for each study or on subgroups, based on the availability of samples. Changes in outcome parameters over time were compared between groups using an independent samples *t* test and/or within groups using a paired *t* test. Non-parametric alternatives (Mann–Whitney *U* test and Wilcoxon signed-rank test) were used for non-normal distributions. For RCT2 and the OLE, sensitivity analyses were performed for vitamin B_6_, vitamin B_12_, folate, choline and Hcy, excluding two patients in the control-active group because of their recent use of vitamin B_12_ injections before baseline, which might have interfered with plasma levels of these parameters. In addition, the impact of sex and age as covariates and as modifiers of the intervention effect on the laboratory parameters of RCT2 was tested using analysis of covariance (ANCOVA) with change from baseline as a dependent variable and baseline as an additional covariate. For these analyses, several parameters were log-transformed to get a distribution closer to the normal distribution.

Statistical analyses were performed using SAS software (SAS Enterprise Guide 4.3 for Windows; SAS Institute, Cary, NC, USA). Data are presented as means ± standard deviation (SD) unless stated otherwise. OLE data are presented according to the intervention received during the double-blind study period of RCT2 (i.e., control-active and active-active). Statistical significance was set at *p* < 0.05 and was not corrected for multiple testing.

## Results

The baseline characteristics of the total study population (RCT1: *n* = 212; RCT2: *n* = 259; RCT3: *n* = 527; and OLE: *n* = 201) are summarized in Table 2. By definition, all studies included a mild or mild-to-moderate AD population (mean MMSE scores of 23.9 [RCT1], 25.0 [RCT2], 19.5 [RCT3] and 25.1 [OLE]), and all subjects were aged 50 years or older (range: 50–95 years; mean ages: 73.7 years [RCT1], 73.8 years [RCT2], 76.7 years [RCT3] and 74.2 years [OLE]).

**Table 2:** Baseline demographics and characteristics of the intention-to-treat study populations

	RCT1		RCT2		OLE		RCT3	
Characteristics	Control (*n* = 106)	Active (*n* = 106)	Control (*n* = 129)	Active (*n* = 130)	Control-Active (*n* = 104)	Active-Active (*n* = 97)	Control (*n* = 262)	Active (*n* = 265)
Male, n (%)	52 (49.1)	54 (50.9)	64 (49.6)	68 (52.3)	52 (50.0)	51 (52.6)	127 (48.5)	126 (47.5)
Age, yr	73.3 (7.8)	74.1 (7.2)	73.2 (8.4)	74.4 (6.9)	73.9 (8.3)	74.5 (6.8)	76.9 (8.2)	76.6 (8.2)
BMI, kg/m^2^	26.2 (3.5)	26.2 (4.8)	26.7 (4.2)	26.1 (4.1)	27.3 (4.2)	26.9 (4.2)	26.6 (4.6)	26.2 (4.5)
Years of education beyond primary school	6.0 (4.0)	5.5 (3.9)	6.0 [0.0–19.0]	6.0 [0.0–20.0]	6.7 (4.7)	6.2 (4.8)	6.4 (3.5)	6.7 (3.6)
Duration of AD since diagnosis, mo	32 [0–1036]^a^	30 [−18 –to 1932]^a^	2 [0–88]	1 [0–70]	2.0 [0.0–88.0]	1.0 [0.0–70.0]	35 (30)	33 (25)
Duration of AD medication use, mo	N/A	N/A	N/A	N/A	N/A	N/A	31 (29)	29 (23)
Nutritional supplement use, *n* (%)^b^								
Vitamins	6 (5.4)	4 (3.5)	12 (9.3)	11 (8.5)	7 (6.7)	8 (8.2)	104 (40.0)	112 (42.4)
Mineral supplements	9 (8.0)	15 (13.3)	12 (9.3)	7 (5.4)	10 (9.6)	4 (4.1)	50 (19.2)	46 (17.4)
General nutrients	11 (9.8)	10 (8.8)	6 (4.7)	8 (6.2)	6 (5.8)	5 (5.2)	29 (11.2)	21 (8.0)
MMSE, total score	24.0 (2.5)	23.8 (2.7)	25.0 (2.8)	24.9 (2.9)	25.1 (3.4)	25.1 (3.3)	19.4 (3.0)	19.5 (3.2)
ApoE ε4 carrier, n (%)								
No	–	–	58 (49.2)	62 (51.2)	46 (48.9)	41 (44.1)	84 (42.0)	87 (39.2)
Yes	–	–	60 (50.8)	59 (48.8)	48 (51.1)	52 (55.9)	116 (58.0)	135 (60.8)
Unknown	–	–	11	9	10	4	62	43

– not done, *AD* Alzheimer’s disease, *ApoE* apolipoprotein E, *BMI* body mass index, *MMSE* Mini Mental State Examination, *N/A* not applicable, *OLE* open-label extension; *RCT* randomized clinical trial

Data are mean (standard deviation) or median [range], unless indicated otherwise

^a^In days instead of months. The value of −18 days represents a protocol deviation; the subject was diagnosed 18 days after baseline assessment

^b^Defined as the number and percentage of subjects using at least one nutritional supplement in the all-subjects-treated population

There were no significant or relevant between-group differences in use of nutritional supplements during the studies, except for the use of vitamin C in RCT3, which was significantly higher in the active group versus the control group (2.3 % [*n* = 6] versus 0 % [*n* = 0]; *p* = 0.030). In RCT3, a large proportion (41.2 %) of subjects used (multi-)vitamins.

### Plasma micronutrients and fatty acids available in Fortasyn Connect

Descriptive statistics for plasma micronutrients and erythrocyte fatty acids available in Fortasyn Connect are presented in Table 3. The results for plasma vitamin E and erythrocyte DHA and EPA have been published in part before [15, 16, 18, 19].

**Table 3:** Descriptive statistics for plasma micronutrients, erythrocyte fatty acids and homocysteine following Fortasyn Connect supplementation

	Control		Active		*p* value
	Baseline	End of study	Baseline	End of study	
Uridine (μM)					
RCT1 (0–24 wk)	3.91 [1.74, 7.06] (72)	4.04 [1.16, 6.72] (68)	3.99 [1.01, 7.67] (77)	4.63 [1.47, 25.94] (72)	0.044*
RCT2 (0–24 wk)	3.5 [0.8, 10.6] (129)	3.5 [0.6, 17.3] (119)	3.6 [0.5, 10.3] (128)	8.6 [1.8, 34.9] (116)	<0.001*
OLE (24–48 wk), C-A, A-A			3.5 [0.6, 17.3] (103), 8.8 [1.8, 28.9] (96)	6.6 [1.6, 22.0] (95), 6.6 [1.4, 22.4] (85)	<0.001* (C-A), <0.001* (A-A)
RCT3 (0–24 wk)	3.6 [0.4, 7.7] (248)	3.2 [0.6, 25.7] (230)	3.6 [1.1, 28.8] (253)	7.4 [1.5, 32.0] (237)	<0.001*
Choline (μM)					
RCT1 (0–24 wk)	8.54 [4.30, 19.90] (72)	8.59 [5.44, 16.00] (67)	9.64 [5.21, 22.50] (75)	11.30 [5.92, 28.10] (73)	<0.001*
RCT2 (0–24 wk)	9.4 [4.4, 18.2] (128)	8.7 [4.5, 18.6] (117)	9.2 [4.5, 18.1] (128)	13.3 [5.8, 28.8] (116)	<0.001*
OLE (24–48 wk), C-A, A-A			8.5 [4.5, 18.6] (101), 12.8 [5.8, 28.8] (96)	14.0 [3.6, 29.4] (91), 14.6 [7.4, 29.2]	<0.001* (C-A), 0.149* (A-A)
Erythrocyte DHA (%)					
RCT1 (0–24 wk)	3.6 [0.7, 7.2] (104)	3.6 [2.0-6.9] (74)	3.6 [2.1, 6.5] (103)	7.0 [1.4-9.2] (73)	<0.001**
RCT2 (0–24 wk)	3.1 [0.0, 5.6] (128)	3.2 [0.7, 6.8] (119)	3.0 [0.3, 5.9] (129)	6.7 [1.3, 8.7] (114)	<0.001*
OLE (24–48 wk), C-A, A-A			3.4 [0.7, 5.8] (103), 6.7 [1.3, 8.7] (94)	6.9 [0.4, 9.1] (93), 6.8 [1.1, 10.8] (87)	<0.001* (C-A), 0.853* (A-A)
RCT3 (0–24 wk)	2.4 [0.0, 6.5] (257)	2.4 [0.2, 4.9] (232)	2.4 [0.0, 8.1] (259)	6.6 [0.9, 10.1] (239)	<0.001
Erythrocyte EPA (%)					
RCT1 (0–24 wk)	0.9 [0.1, 3.4] (104)	0.9 [0.1, 3.6] (74)	1.0 [0.1, 2.8] (103)	1.8 [0.0, 3.1] (73)	<0.001**
RCT2 (0–24 wk)	0.8 [0.0, 3.3] (128)	0.8 [0.2, 2.7] (119)	0.8 [0.0, 2.9] (129)	1.6 [0.3, 4.0] (114)	<0.001*
OLE (24–48 wk), C-A, A-A			0.8 [0.3, 2.7] (103), 1.6 [0.6, 4.0] (94)	1.7 [0.1, 4.8] (93), 1.6 [0.5, 3.6] (87)	<0.001* (C-A), 0.730* (A-A)
RCT3 (0–24 wk)	0.4 [0.0, 3.6] (257)	0.5 [0.0, 2.0] (232)	0.4 [0.0, 2.6] (259)	1.2 [0.0, 4.3] (239)	<0.001
Plasma DHA (%)					
RCT1 (0–24 wk)	1.8 [0.8, 4.9] (91)	1.9 [1.0, 5.4] (54)	1.9 [0.8, 6.2](91)	3.8 [1.2, 6.6](66)	<0.001**
RCT2 (0–24 wk)	1.7 [0.7, 3.4] (129)	1.6 [0.6, 3.4] (119)	1.7 [0.7, 4.3] (129)	4.7 [1.3, 7.7] (115)	<0.001
OLE (24–48 wk), C-A, A-A			1.6 [0.8, 3.4] (103), 4.7 [1.3, 7.7] (95)	4.9 [1.1, 7.2] (95), 4.9 [1.4, 9.4] (87)	<0.001* (C-A), 0.357* (A-A)
Plasma EPA (%)					
RCT1 (0–24 wk)	0.8 [0.2, 4.0] (91)	0.8 [0.4, 3.4] (54)	0.8 [0.2, 2.9] (91)	1.4 [0.4, 3.8] (66)]	<0.001**
RCT2 (0–24 wk)	0.8 [0.2, 3.9] (129)	0.7 [0.0, 2.8] (119)	0.8 [0.3, 4.5] (129)	1.7 [0.4, 4.9] (115)	<0.001*
OLE (24–48 wk), C-A, A-A			0.7 [0.0, 2.8] (103), 1.7 [0.4, 4.9] (95)	1.9 [0.4, 5.4] (95), 1.7 [0.4, 5.0] (87)	<0.001* (C-A), 0.688* (A-A)
Folate (nM)					
RCT1 (0–24 wk)	15.04 [0.31, 66.70] (71)	16.45 [2.91, 154.8] (63)	14.55 [2.85, 68.46] (76)	44.40 [14.02, 182.0] (66)	<0.001*
RCT2 (0–24 wk)	12.6 [2.4, 45.3] (129)	13.5 [2.7, 45.3] (119)	12.3 [3.6, 45.3] (128)	37.3 [12.2, 45.3] (115)	<0.001*
OLE (24–48 wk), C-A, A-A			13.5 [4.8, 45.3] (103), 37.3 [12.2, 45.3] (95)	37.2 [16.2, 83.4] (95), 39.4 [6.4, 77.1] (87)	<0.001* (C-A), 0.405* (A-A)
Vitamin B_12_ (pM)					
RCT1 (0–24 wk)	282.0 [71.0, 971.0] (71)	266.5 [68.0, 1265.0] (66)	250.0 [87.0, 676.0] (75)	311.0 [97.0, 856.0] (71)	<0.001*
RCT2 (0–24 wk)	300.0 [90, 1476] (129)	308.0 [156, 1476] (119)	289.5 [127, 1476] (128)	322.0 [177, 1476] (116)	<0.001*
OLE (24–48 wk), C-A, A-A			310.0 [163, 1476] (103), 323.0 [117, 1476] (96)	328.0 [166, 1476] (95), 312.0 [154, 1476] (87)	0.030* (C-A), <0.001* (A-A)
Vitamin B_6_ (nM)					
RCT2 (0–24 wk)	45.6 [11.5, 182.3] (45)	42.6 [9.4, 128.0] (41)	37.2 [13.5, 257.2] (37)	59.5 [27.1, 377.4] (36)	<0.001*
OLE (24–48 wk), C-A, A-A			43.0 [12.9, 128.0] (38), 60.3 [27.1, 377.4] (33)	84.9 [44.8, 173.8] (17), 76.8 [33.0, 121.9] (13)	<0.001* (C-A), 0.244* (A-A)
Vitamin E (μM)					
RCT1 (0–24 wk)	31.9 [12.3, 66.2] (104)	30.9 [14.3, 78.5] (74)	33.1 [19.2, 75.2] (104)	39.6 [13.1, 83.6] (74)	<0.001**
RCT2 (0–24 wk)	32.0 [9.2, 70.6] (129)	33.2 [14.4, 61.6] (119)	32.1 [18.0, 71.4] (129)	41.6 [25.7, 73.6] (116)	<0.001*
OLE (24–48 wk), C-A, A-A			32.8 [14.4, 61.6] (103), 41.7 [25.7, 73.6] (96)	40.2 [20.7, 72.4] (93), 41.5 [20.1, 68.6] (88)	<0.001* (C-A), 0.319* (A-A)
RCT3 (0–24 wk)	29.9 [8.5, 99.8] (255)	30.6 [6.0, 78.4] (233)	29.6 [1.8, 84.3] (260)	38.5 [17.1, 109.4] (239)	<0.001
Selenium (μM)					
RCT1 (0–24 wk)	1.1 [0.6-1.8] (73)	1.0 [0.6-1.5] (68)	1.1 [0.6, 2.1] (75)	1.3 [0.7, 2.0] (72)	<0.001**
RCT2 (0–24 wk)	1.1 [0.3, 1.6] (129)	1.1 [0.5, 1.8] (119)	1.1 [0.6, 1.9] (129)	1.4 [0.7, 2.0] (116)	<0.001*
OLE (24–48 wk), C-A, A-A			1.1 [0.5, 1.8] (103), 1.4 [0.7, 2.0] (96)	1.3 [0.8, 1.7] (15), 1.3 [1.1, 1.5] (15)	0.007* (C-A), 0.017* (A-A)
Homocysteine (μM)					
RCT1 (0–24 wk)	11.7 [5.0, 83.0] (104)	12.0 [6.8, 42.1] (74)	12.5 [6.6, 36.7] (104)	9.7 [4.0, 19.1] (74)	<0.001**
RCT2 (0–24 wk)	11.7 [3.9, 28.3] (129)	13.4 [3.3, 38.8] (119)	12.1 [4.4, 37.3] (129)	10.5 [2.3, 20.3] (116)	<0.001*
OLE (24–48 wk), C-A, A-A			13.9 [3.3, 38.8] (103), 10.3 [2.3, 20.3] (96)	9.2 [4.7, 17.1] (93), 9.3 [4.3, 19.0] (86)	<0.001* (C-A), <0.001* (A-A)
RCT3 (0–24 wk)	10.6 [3.9, 100.5] (256)	11.0 [3.5, 102.5] (234)	10.4 [12.8, 50.5] (260)	9.8 [2.0, 46.1] (239)	0.004*

*A-A* active-active, *C-A* control-active; *DHA* docosahexaenoic acid; *EPA* eicosapentaenoic acid; *OLE* open-label extension; *RCT* randomized clinical trial

Data are median [min, max] (*n*) for all parameters and RCTs to present comparable data

*Mann–Whitney *U* test, change from baseline at week 24, control vs. active (RCT1, RCT2 and RCT3); Wilcoxon signed-rank test, 24 weeks vs. 48 weeks within control-active (C-A) and active-active (A-A) group (OLE)

**Independent samples *t* test, change from baseline at week 24, control vs. active

Plasma levels of uridine, choline, folate, vitamins B_6_ and B_12_, selenium and vitamin E were all significantly increased in the active versus control group from baseline to week 24 in RCT1, RCT2 and RCT3 (all *p* < 0.001, except for uridine in RCT1 [*p* = 0.044], Mann–Whitney *U* test) (Table 3). During the OLE, plasma levels of these parameters were significantly increased in the control-active group from week 24 to week 48 (i.e., after switching to the active product upon entry into the OLE study) (all *p* < 0.001, Wilcoxon signed-rank test). In addition, plasma levels remained consistently elevated in the active-active group during the OLE, except for plasma levels of uridine, vitamin B_12_ and selenium, which significantly decreased in the active-active group from week 24 to week 48 (Table 3). Despite the latter, however, there was a significant overall increase in plasma uridine and selenium, but not in vitamin B_12_, from baseline to week 48 in the active-active group (*p* < 0.001, Wilcoxon signed-rank test).

In line with the above-described data, the percentages DHA and EPA of total fatty acids in both plasma and the erythrocyte membrane also were significantly increased in the active versus control group from baseline to week 24 in RCT1, RCT2 and RCT3 (*p* < 0.001, *t* test [RCT1] or Mann–Whitney *U* test [RCT2 and RCT3]) (Table 3, Fig. 1). During the OLE, levels remained consistently elevated in the active-active group and significantly increased in the control-active group from week 24 to week 48 (*p* < 0.001, Wilcoxon signed-rank test) (Table 3, Fig. 1). The results for n-3 LC-PUFA in plasma and erythrocytes in RCT2, RCT3 and the OLE were all in line with the results for the individual fatty acids DHA and EPA. Comparable results were obtained in the sensitivity analyses of plasma choline, folate and vitamins B_6_ and B_12_.

A modifying intervention effect of sex on log-transformed percentage of EPA in erythrocyte membrane was found in RCT2 (*p* = 0.021, ANCOVA). Post hoc analyses revealed significantly increased percentages of EPA in the active versus control group from baseline to week 24 for both men and women, but the effect was larger in women than in men (treatment effects of 0.446 for women [*p* < 0.001, *t* test] and 0.343 for men [*p* < 0.001, *t* test]). No other effects of sex were found. Age was neither a significant covariate nor a significant intervention modifier for any of the micronutrients or fatty acids in RCT2.

### Plasma markers of inflammation and oxidative stress

Plasma markers of inflammation (CRP, IL-1β, IL-6 and IL-10) and oxidative stress (8-isoprostane and MDA) were measured at baseline and week 24 in subgroups of the RCT1 study population. No statistically significant between-group differences were observed for the change in any of these parameters (Table 4).

**Table 4:** Descriptive statistics for plasma markers of inflammation and oxidative stress in randomized clinical trial 1

Marker	Baseline	Week 24	*p* value
CRP (mg/L)			
Control	1.75 [0.00, 66.90] (44)	1.80 [0.20, 58.90] (45)	0.686*
Active	2.00 [0.10, 17.40] (47)	1.80 [0.10, 16.40] (47)	
IL-1β (pg/ml)			
Control	0.24 [0.20, 0.66] (25)	0.24 [0.20, 1.48] (23)	0.309*
Active	0.24 [0.20, 0.68] (30)	0.24 [0.20, 0.82] (25)	
IL-6 (pg/ml)			
Control	2.67 [0.23, 108.3] (26)	2.59 [0.93, 6.63] (23)	0.733*
Active	2.65 [0.26, 8.65] (31)	3.20 [0.31, 10.62] (25)	
IL-10 (pg/ml)			
Control	0.91 [0.40, 2.63] (25)	1.01 [0.31, 1.72] (23)	0.799*
Active	1.06 [0.05, 2.88] (32)	1.00 [0.11, 2.68] (27)	
8-isoprostane (pg/ml)			
Control	17.78 [4.55, 80.85] (25)	22.12 [8.81, 38.40] (23)	0.071*
Active	19.06 [6.01, 36.32] (30)	17.66 [5.73, 35.66] (25)	
MDA (μmol/L)			
Control	1.23 (0.53) [45]	1.45 (0.57) [45]	0.786**
Active	1.22 (0.56) [47]	1.48 (0.54) [47]	

*CRP* C-reactive protein, *IL* interleukin, *MDA* malondialdehyde

Data are mean (standard deviation) [*n*] or median [min, max] (*n*)

*Mann–Whitney *U* test, change from baseline at week 24, control vs. active

**Independent samples *t* test, change from baseline at week 24, control vs. active

### Other micronutrients and fatty acids

The results for plasma Hcy have been published in part before [15, 16, 18, 19]. They showed significantly decreased levels in the active group versus the control group from baseline to week 24 in RCT1, RCT2 and RCT3 (*p* < 0.001 [RCT1 vs. RCT2] and *p* = 0.004 [RCT3], Mann–Whitney *U* test). During the OLE, plasma Hcy levels were significantly decreased from week 24 to week 48 in the control-active group (*p* < 0.001, Wilcoxon signed-rank test) and continued to decrease within the active-active group (*p* < 0.001, Wilcoxon signed-rank test).

The percentage DPA of total fatty acids in the erythrocyte membrane was significantly decreased in the active versus control group from baseline to week 24 in RCT2 and RCT3 (*p* < 0.001, Mann–Whitney *U* test). During the OLE, levels remained consistently decreased in the active-active group and significantly decreased in the control-active group from week 24 to week 48 (*p* < 0.001, Wilcoxon signed-rank test).

For plasma levels of uracil, uric acid, pre-albumin (RCT1), vitamin D and DPA (RCT2) and albumin (RCT1 and RCT2), no statistically significant between-group differences were observed for the change in any of these parameters (data not shown). Cytidine was not detectable in the plasma samples using the current laboratory method.

## Discussion

Circulating levels of micronutrients and fatty acids, including uridine, selenium, folate, vitamin B_12_, vitamin E, vitamin C, DHA and EPA, are reported to be decreased in the AD population and can be increased by 12–48-week oral supplementation with Souvenaid. All micronutrients and fatty acids present in this specific nutrient combination (containing Fortasyn Connect) show increased plasma (and erythrocyte for fatty acids) concentrations after 24 weeks of daily use of active product versus control product in patients with mild and mild-to-moderate AD, except for vitamin C, which could not be reliably measured. Most nutrients remain unchanged during prolonged intake for another 24 weeks. Data derived from RCT1 on vitamin E and percentage DHA and EPA of total fatty acids in erythrocyte membrane suggest that circulating levels are increased already at week 6 and that a plateau is reached within 12 weeks of daily intake (shown for DHA in Fig. 1, upper left panel). This is supported by studies showing rising plasma levels within hours of administration of, for example, uridine monophosphate (UMP) [11], vitamin C [34] and vitamin E [35]. Together, these findings suggest that other micronutrients investigated in this study might follow a similar pattern of increase, as shown for the percentage DHA in the erythrocyte membrane (Fig. 1).

All components of Fortasyn Connect are precursors or cofactors required for neuronal membrane formation [36]. Increasing availability of all substrates for the formation of phosphatidylcholine (PC) and phosphatidylethanolamine (PE) is important to enable neuronal membrane synthesis, ultimately support synapse maintenance and/or promote synapse formation [13, 37]. Increases in the substrates could underlie the improvement in memory domain scores that were found in the trials in drug-naïve patients with mild AD after 12 weeks (RCT1), 24 weeks (RCT1, RCT2 and OLE) and the exploratory data for the additional 24 weeks (OLE) [15–17]. No change in cognition was found, however, after 24 weeks in the clinical trial in patients with mild-to-moderate AD or in those taking AD medication (RCT3) [18]. These results suggest that early intervention is likely key to an effect on cognition.

This is the first study, to the best of our knowledge, in which the effects of sustained dietary intake of UMP on plasma uridine levels in humans are reported. We showed previously that intake of a single serving of 625 mg of UMP by healthy human subjects resulted in peak plasma uridine concentration of 14.6 μmol/L at 1 hour after intake [11]. The present data indicate that daily intake of the same UMP-containing intervention for 24 and 48 weeks increases fasting (RCT1) and non-fasting (RCT2, RCT3 and OLE) plasma uridine in people with AD. This new observation is particularly relevant in view of recent studies which indicate that plasma uridine [10, 38] and cerebrospinal fluid (CSF) uridine [39, 40] levels are lower in AD than in controls. Other investigations indicated that circulating and brain levels of DHA and choline are lower in people with AD than in controls [41–44]. The results of the present study indicate that, in addition to uridine, the investigational product increases circulating DHA and choline, a finding in line with other intervention studies showing that supplemental dietary DHA and choline increase their circulating levels in various populations [45–48]. Uridine, together with DHA and choline, are the rate-limiting precursors via the Kennedy pathway for the synthesis of phospholipids in neuronal membranes, which are depleted in AD [49, 50]. As combined dietary enrichment of these nutrients has been reported to promote the synthesis of brain phospholipids, hippocampal dendritic spines and synaptic proteins, all prerequisites for synaptogenesis [51] and repletion of DHA, choline and uridine may therefore contribute to counteract the characteristic synaptic loss in AD. Tissue target levels of DHA, choline and uridine are not well defined for the general population; however, given their role in neuronal membrane synthesis, people with AD likely require higher levels to compensate for membrane loss. The present study indicates that the investigated intervention is efficacious in enhancing circulating levels of these nutrients in AD.

In RCT1, markers of inflammation and oxidative stress were measured as exploratory outcomes to investigate whether the n-3 LC-PUFA and the antioxidants vitamins C and E had a direct effect on inflammation or oxidative stress, respectively. No changes were found, however, in plasma markers of inflammation (CRP, IL-1β, IL-6 and IL-10) or oxidative stress (8-isoprostane and MDA) in a subgroup of the RCT1 population after 24 weeks of intake of this specific nutrient combination compared with a control product. Similarly to Freund-Levi et al. [52], who did not find an effect on inflammatory markers in either plasma or CSF, we found no effects on any of the markers of inflammation, even though the current intervention contained two to three times greater amounts of DHA and EPA. In some studies in which researchers did find an effect of antioxidant supplements on markers of oxidative stress in patients with AD contained much higher doses of, for example, vitamin E [53] (3500 % versus 400 % of RDA) than those used in the present study, whereas others used comparable dosages [54] (200 % of RDA). Differences still exist, however, in the timing and duration of the intervention and in the method used to measure oxidative stress. These results suggest that changes in cognition found in the trials and the OLE were not due to mediation of inflammation or oxidative stress.

As expected on the basis of equal amounts of vitamin D, protein and energy contained in the active and control product, plasma levels of prealbumin (RCT1), vitamin D (RCT2) and albumin (RCT1 and RCT2) were not altered by intake of this specific nutrient combination.

Plasma Hcy levels were significantly decreased after 24 weeks in the active group and continued to decrease with prolonged intake. This is in line with the observed increases in vitamin B_6_ and B_12_ and folic acid levels. Increased B-vitamin levels and decreased Hcy levels enhance methylation capacity, thereby increasing PE to PC by the PE–N-methyltransferase pathway in the liver. This leads to increased availability of choline and DHA-rich PC for (neuronal) membrane synthesis [24, 55]. In addition, decreased Hcy levels may indicate improved vascular health. Previous animal studies showed increased cerebral blood flow in a 12-month-old mouse model of AD fed this specific nutrient combination compared with a control diet [56].

The increase in percentage EPA of total fatty acids in erythrocyte membrane after 24 weeks in the active group in RCT2 was greater in women than in men. This could be a true sex difference, as Burdge et al. [57, 58] showed a higher conversion rate of α-linolenic acid to EPA (and DHA) in young women than in young men, or it could be due to differences in body weight. Flock et al. [59] showed that body weight significantly improved prediction of treatment response of EPA + DHA supplements on erythrocyte membrane content of EPA compared with dose only. However, a trend for an increased treatment response in women compared with men was found in this same study while already correcting for body weight, suggesting an effect of sex independent from body weight. Despite the uncertainty about the underlying mechanism, the incorporation of EPA in erythrocyte membrane is apparently greater in our population of elderly women with AD than in men, although it was significant in both sex groups.

DPA erythrocyte (but not plasma [RCT2]) concentrations were decreased from baseline to week 24 in the active group compared with the control group in RCT2 and RCT3, and it reached a plateau in the OLE at week 48. Possibly, the increasing availability of the two other major n-3 LC-PUFAs, DHA and EPA, reduces the incorporation of DPA in erythrocytes while plasma levels remain stable [60].

The data presented in this article on the increased levels of multiple nutrients and fatty acids are generally in line with known kinetics of single nutrients, which assures that there is no relevant interaction in absorption. We found consistent results over three large RCTs when we evaluated the ITT population, representing patients with both mild and mild-to-moderate AD and including patients using AD medication as well as drug-naïve patients. However, we report on exploratory analyses, and none of our outcome measures were primary endpoints of the studies. Furthermore, it should be noted that the present data refer only to circulating levels, whereas the nutrients should have their effect in the brains of patients with AD. With the current data, we cannot confirm whether these nutrients or their metabolites cross the blood–brain barrier and have an effect on synapse synthesis and maintenance. Evidence exists, however, to support the proposed mechanism of action. On the basis of tracing studies done with positron emission tomography (PET), we know that at least the precursors for the Kennedy pathway (i.e., choline, DHA and uridine) do reach the brain within hours of administration in animals and/or healthy humans [61–63]. For instance, Umhau et al. [62] showed that, based on PET measurements 60 minutes following administration of [1-^11^C]DHA, the incorporation rate of DHA could be calculated in healthy human volunteers. Additionally, in a subset of the study population of RCT2, additional electroencephalographic measurements were performed to assess underlying synaptic function. We found preserved organization of brain networks in patients with mild AD within 24 weeks, compared with a control product, hypothetically counteracting the progressive network disruption over time in AD [64]. Currently, a study of drug-naïve patients with mild AD is ongoing to investigate the effect of this specific nutrient combination on brain phospholipid metabolism by phosphorus magnetic resonance spectroscopic imaging (MRSI) [65]. In contrast to the present study, the MRSI study will provide more direct evidence on the extent to which the nutrients and their metabolites can affect neuronal membrane turnover and influence synaptic function.

## Conclusions

These data show that circulating levels of nutrients, known to be decreased in the AD population, can be increased in patients with mild and mild-to-moderate AD by 24–48-week oral supplementation with Souvenaid. Uptake is observed within 6 weeks, and a plateau phase is reached for most nutrients during prolonged intake, thus increasing the availability of precursors and cofactors necessary for the formation and function of neuronal membranes and synapses for the brain. This adds to the rationale of using oral supplementation of micronutrients to replenish nutritional deficits in patients with AD.

## Additional file

Additional file 1:
**List of independent ethics committees and institutional review boards.**

## Abbreviations

A-A: Active-active
AD: Alzheimer’s disease
ANCOVA: Analysis of covariance
ApoE: Apolipoprotein E
BMI: Body mass index
C-A: Control-active
CRP: C-reactive protein
CSF: Cerebrospinal fluid
DHA: Docosahexaenoic acid
DPA: Docosapentaenoic acid
EPA: Eicosapentaenoic acid
Hcy: Homocysteine
HPLC: High-performance liquid chromatography
IL: Interleukin
ITT: Intention to treat
MDA: Malondialdehyde
MMSE: Mini Mental State Examination
MRSI: Magnetic resonance spectroscopic imaging
n-3 LC-PUFA: n-3 long-chain polyunsaturated fatty acids
OLE: Open-label extension
PC: Phosphatidylcholine
PE: Phosphatidylethanolamine
PET: Positron emission tomography
RCT: Randomized clinical trial
RDA: Recommended dietary allowance
SD: Standard deviation
TE: Tocopherol equivalents
UMP: Uridine monophosphate
