# Supplementary material for: Effects of Souvenaid on plasma micronutrient levels and fatty acid profiles in mild and mild-to-moderate Alzheimer’s disease
Source: Alzheimers Res Ther. 2015 Jul 24;7(1):51. doi: 10.1186/s13195-015-0134-1 (PMC4513634; doi:10.1186/s13195-015-0134-1)
Supplement: Additional file 1: — List of independent ethics committees and institutional review boards. [file 13195_2015_134_MOESM1_ESM.pdf]

List of Independent Ethics Committees or Institutional Review Boards

Souvenir I

The Netherlands:
- VU medical centre, Amsterdam
- Radboud University medical centre, Nijmegen
- Academic Hospital Maastricht, Maastricht
- Jeroen Bosch Hospital, ’s-Hertogenbosch
- University Medical Centre Utrecht, Utrecht
- Medical Centre Alkmaar, Alkmaar
- University Medical Centre Groningen, Groningen
- Gelderse Vallei Hospital, Ede
- Tergooiziekenhuizen, Blaricum
- Diaconessenhuis Leiden, Leiden
- Amphia Hospital, Breda

Belgium:
- University Hospital Gasthuisberg, Leuven
- AZN Middelheim, Antwerpen
- Virga Jess kliniek, Hasselt
- Universitair Ziekenhuis Gent, Gent
- Heilig Hart ziekenhuis, Roeselare
- Sint Andries ziekenhuis, Tielt

Germany:
- Ethikkommission der Fakultät für Medizin der Technischen Universität München, München
- Ethikkommission der Sächsische Landesärztekammer, Dresden
- Ethikkommission Medizinische Fakultät Carl Gustav Carus der Technische Universität Dresden, Dresden
- Ethikkommission der Medizinischen Fakultät Heidelberg, Heidelberg
- Ethik-Kommission der Ärtzekammer Hamburg, Hamburg
- Ethik-Kommission der Georg-August Universität Göttingen, Göttingen
- Ethik-Kommission der Medizinischen Fakultät der Ruhr-Universität Bochum, Bochum
- Ethikkommission der Ärztekammer Nordrhein, Düsseldorf
- Ethik-Kommission der Ärztekammer Schleswig-Holstein, Bad Segeberg
- Ethik-Kommission bei der Landesärtztekammer Hessen, Frankfurt am Main
- Ethik-Kommission der Bayerischen Landesärtztekammer, München
- Ethik-Kommission der Georg-August Universität Göttingen, Göttingen
- Ethik-Kommission der Medizinischen Fakultäat der Ruhr-Universität Bochum, Bochum
- Ethik-Kommission der Ärztekammer Nordrhein, Düsseldorf

United Kingdom:
- Southampton & South West Hampshire REC, Southampton
- Bath Local Research Ethics Committee
- Swindon Research Ethics Committee
- Bradford Research Ethics Committee
- Cumbria and Lancashire Ethics Committee

United States:
- Institutional Review Board Saint Louis University Care, St. Louis

Souvenir II

The Netherlands
For central approval
- Medisch Ethische Toetsingscommissie VUmc, Amsterdam

For assessment of local feasibility
- Medisch Ethische Commissie azM/UM, Maastricht
- Commissie Mensgebonden Onderzoek regio Arnhem-Nijmegen
- Medisch Ethische Toetsingscommissie Catharina Ziekenhuis, Eindhoven
- Medisch Ethische Toetsingscommissie Amphia, Breda
- Wetenschapsbureau Jeroen Bosch Ziekenhuis, Den Bosch
- LAWO Orbis Medisch Centrum (Lokale Adviesgroep Wetenschappelijk Onderzoek), Sittard
- Regionale Toetsingscommissie Patiëntgebonden Onderzoek, Leeuwarden
- Medisch Ethische Toetsingscommissie Tergooiziekenhuizen, Blaricum

Belgium
For central approval
- Commissie Medische Ethiek ZNA/O.C.M.W., Antwerp

For local positive opinion
- Comité voor Medische Ethiek, Sint-Andriesziekenhuis Tielt
- Medische Ethische Commissie H. Hartziekenhuis, Roeselare
- Ethisch Comité vzw Emmaüs, AZ Sint-Maarten, Mechelen
- Ethisch Comité Sint-Trudoziekenhuis, Sint-Truiden

Germany
For central approval by each EC
- Ethik-Kommission der Universität Ulm
- Ethik-Kommission der Ärztekammer Nordrhein
- Ethik-Kommission der sächsischen Landesärztekammer
- Ethik-Kommission der Ärztekammer Westfalen-Lippe und der Medizinischen Fakultät der Westfälischen Wilhelms-Universität Münster
- Ethikkommission der medizinischen Fakultät Heidelberg
- Ethik-Kommission der Bayerischen Landesärztekammer
- Ethik-Kommission der Medizinischen Fakultät der Universität zu Köln Forum

Spain
For central approval by each EC
- Comité ético de investigación clínica. Hospital de la Santa Creu i Sant Pau, Barcelona
- Comité ético de investigación clínica. Hospital Clínico San Carlos, Madrid
- H.Clinic I Provincial EC Agencia de Ensayos Clínicos, Hospital Clinic de Barcelona.
- Hospital Virgen Arrixaca EC, Murcia

Italy
For central approval by each EC
- Comitato Etico Fondazione Ospedale Maggiore, Milan
- Comitato Etico Aziende Sanitarie Umbria
- Comitato Etico dell'Università Cattolica del Sacro Cuore, Rome
- Comitato Etico dellâ´Azienda Ospedaliera Universitaria s. Martino di Genova

France
For central approval
- CPP sud-ouest, Toulouse

Open-label extension

The Netherlands
For central approval
- Independent Review Board Nijmegen (IRBN), Nijmegen

For assessment of local feasibility
- Medisch Ethische Toetsingscommissie VUmc, Amsterdam
- Medisch Ethische Commissie azM/UM, Maastricht
- Commissie Mensgebonden Onderzoek regio Arnhem-Nijmegen
- Medisch Ethische Toetsingscommissie Catharina Ziekenhuis, Eindhoven
- Medisch Ethische Toetsingscommissie Amphia, Breda
- Wetenschapsbureau Jeroen Bosch Ziekenhuis, Den Bosch
- LAWO Orbis Medisch Centrum (Lokale Adviesgroep Wetenschappelijk Onderzoek), Sittard
- Regionale Toetsingscommissie Patiëntgebonden Onderzoek, Leeuwarden
- Medisch Ethische Toetsingscommissie Tergooiziekenhuizen, Blaricum

Belgium
For central approval
- Commissie Medische Ethiek ZNA/O.C.M.W., Antwerp

For local positive opinion
- Comité voor Medische Ethiek, Sint-Andriesziekenhuis Tielt
- Medische Ethische Commissie H. Hartziekenhuis, Roeselare
- Ethisch Comité vzw Emmaüs, AZ Sint-Maarten, Mechelen
- Ethisch Comité Sint-Trudoziekenhuis, Sint-Truiden

Germany
For central approval by each EC
- Ethik-Kommission der Universität Ulm
- Ethik-Kommission der Ärztekammer Nordrhein
- Ethik-Kommission der sächsischen Landesärztekammer
- Ethikkommission der medizinischen Fakultät Heidelberg
- Ethik-Kommission der Bayerischen Landesärztekammer

Spain
For central approval by each EC
- Comité ético de investigación clínica. Hospital de la Santa Creu i Sant Pau, Barcelona
- Comité ético de investigación clínica. Hospital Clínico San Carlos, Madrid
- H.Clinic I Provincial EC Agencia de Ensayos Clínicos, Hospital Clinic de Barcelona.

Italy
For central approval by each EC
- Comitato Etico Fondazione Ospedale Maggiore, Milan
- Comitato Etico Aziende Sanitarie Umbria
- Comitato Etico dellâ´Azienda Ospedaliera Universitaria s. Martino di Genova

France
Central approval
- CPP sud-ouest, Toulouse

S-Connect

United States

- Quorum Review IRB as the central Ethics Committee for sites 013, 040, 049, 022, 005, 034, 028, 047, 056, 025, 045, 051, 052, 041, 053, 004, 042, 024, 050, 021, 017, 039, 046, 029, 002, 057, 023,032, 036, 037, 043, 048, 020, 027, 033 (see also chapter 2 for detailed site information).
- For site 011: Medical College of Wisconsin, IRB Human Research Review Committee
- For site 014: University of Kansas Medical Center, Human Subjects Committee
- For site 003: Indiana University, IRB
- For site 008: University of Pennsylvania, IRB
- For site 035: University of Kentucky, Medical IRB
- For site 044: Drexel University College of Medicine, IRB
- For site 038: Medical University of South Carolina, IRB
- For site 006: Saint Louis University, IRB (Biomedical)
- For site 011: Oregon Health and Science University, IRB
- For site 012: University of Florida, IRB
- For site 007: University of Texas Health Science Center, IRB
- For site 001: Rush University Medical Center, IRB
- For site 016: Wake Forest University Health Sciences, IRB

Study centres S-Connect

Principal investigators:	*Site, USA*
– 001 - Shah Raj C	Rush University Medical Center, Chicago, IL
– 002 - Mulroy Amy E.	Clinical Trials of Texas Inc., San Antonio, TX
– 003 - Farlow Martin R	Dept of Neurology, Indiana Alzheimer Disease Center Indianapolis, IN
– 004 - Hyman Lawrence R	Howard Behavioral Health Inc. Columbia Medical Center, Columbia, MD
– 005 - Schwartz Matthew T	Behavioral Medical Research of Brooklyn, Brooklyn, NY
– 006 - Morley John E	Saint Louis University Medical Center, St. Louis, MO
– 007 - Royall Donald R.	University of Texas Health Science Center, San Antonio, TX
– 008 - Huege Steven F	Penn Memory Center University of Pennsylvania, Philadelphia, PA
– 010 - Antuono Piero G	Medical College of Wisconsin, Milwaukee, WI
– 011 - Quinn Joseph	Oregon Health and Science University Aging and Alzheimer Disease Center, Portland, OR
– 012 - Raj Ashok	Byrd Alzheimer's Institute Tampa, FL
– 013 - Baker Matthew J.	Collier Neurologic Specialists, Naples, FL
– 014 - Burns Jeffrey M.	University of Kansas Medical Center - Department of Neurology, Kansas City, KS
– 016 - Sink Kaycee	Kylynych Center for Memory & Cognition Research Wake Forest University Health Sciences, Winston-Salem, NC
– 017 - Kudrow David	David Kudrow MD, Santa Monica, CA
– 020 - Shua-Haim Joshua R	Alzheimer's Research Corporation, Manchester, NY
– 021 - Knesevich Mary Ann	Mary Ann University Hills Clinical Research, Irvine, TX
– 022 - Breving Joel T	Behavioral Medical Research of Staten Island, Staten Island, NY
– 023 - Omidvar Omid	Collaborative Neuroscience Network, Garden Grove, CA
– 024 - Jones Beverly Nicholas	Clinical Trials of America Inc, Winston-Salem, NC
– 025 - Duboff Eugene	Radiant Research, Denver, CO
– 027 - Tuten Cindy	Clinical Study Center of Asheville LLC, Asheville, NC
– 028 - Cohen Steven R.	Suncoast Neuroscience Associates INC, St. Petersburg, FL
– 029 - McElveen William Alvin	Bradenton Research Center, Bradenton, FL
– 032 - Randhawa Surinder K.	Lynn Health Science Institute, Oklahoma City, OK
– 033 - Weiss Thomas R.	Radiant Research, San Antonio San Antonio, TX

– 034 - Chang Fen-Lei F.	Fort Wayne Neurological Center, Fort Wayne, IN
– 035 - Jicha Gregory A.	University of Kentucky Medical Center Sanders-Brown Center on Aging, Lexington, KY
– 036 - Rappaport Stephen A.	Agewell, Indianapolis, IN
– 037 - Sadowsky Carl H.	Premiere Research Institute, West Palm Beach, FL
– 038 - Mintzer Jacobo	Medical University of South Carolina, N. Charleston, SC
– 039 - Kyle Michael L.	Radiant Research Inc., Chicago, IL
– 040 - Bari Mohammed A.	Synergy Clinical Research Center, National City, CA
– 041 - Goldstein Jerome	San Francisco Clinical Research Center, San Francisco, CA
– 042 - Jariwala Arvind N.	Wake Research Associates LLC, Raleigh, NC
– 043 - Sergi Paul A	Beacon Clinical Research LLC, Brockton, MA
– 044 - Lippa Carol F.	Drexel University College of Medicine Department of Neurology, Philadelphia, PA
– 045 - Farmer Mildred V.	Meridien Research, Brooksville, FL
– 046 - Lefebvre Gigi Claire	Meridien Research, St. Petersburg, FL
– 047 - Cornacchione Mario	Northeastern Pennsylvania Memory & Alzheimer's Center, Plains, PA
– 048 - Shepherd Timothy S	Shepherd Healthcare, Lewisville, TX
– 049 - Bernstein, Allan L	Radiant Research, Inc., Santa Rosa, CA
– 050 - Keating, Willem J	North Georgia Premier Research, Dawsonville, Georgia
– 051 - Forchetti, Conceta	NSI Research, Elk Grove Village, IL
– 052 - Gfeller, Eduard	Florida Clinical Research Centers, Maitland, FL
– 053 - Hogancamp, Willem	Innovative Clinical Concepts, Paducah, KY
– 056 - Downing, Michael	FS Trials, Dallas, TX
– 057 - Naslund, Patricia K.	Raleigh Neurology Associates, Raleigh, NC
